# Synthesis of a novel hydrazone-based compound applied as a fluorescence turn-on chemosensor for iron(iii) and a colorimetric sensor for copper(ii) with antimicrobial, DFT and molecular docking studies†

**DOI:** 10.1039/d3ra04364a

**Published:** 2023-08-09

**Authors:** Sharmin Akther Rupa, Md Abdul Majed Patwary, William Emmanuel Ghann, Adams Abdullahi, A. K. M. Royhan Uddin, Md. Mayez Mahmud, Md. Aminul Haque, Jamal Uddin, Mohsin Kazi

**Affiliations:** a Department of Chemistry, Comilla University Cumilla-3506 Bangladesh mamajedp@gmail.com; b Center for Nanotechnology, Department of Natural Sciences, Coppin State University Baltimore USA; c Tokushima University, Faculty of Pharmaceutical Science Tokushima Shi 770-0026 Japan; d Department of Chemistry, Jagannath University Dhaka-1100 Bangladesh; e Department of Pharmaceutics, College of Pharmacy, King Saud University P.O. Box 2457 Riyadh 11451 Saudi Arabia

## Abstract

Hydrazone–hydrazide-based linkers perform a crucial role in environmental as well as biological fields. Such linkers are employed to detect exact metal ions at a minute level; hence, numerous probes are available. Even though thiophene-based molecules have a unique position in the medicinal arena, only very few chemosensors are reported based on such a moiety. In this current work, a novel hydrazide–hydrazone-based fluorogenic molecule 5-bromo-2-hydroxy-*N*′-[(1*E*)-1-(thiophen-2-yl)ethylidene]benzohydrazide (L) has been successfully designed and synthesized. The sensing studies of L demonstrated a ratio metric as well as turn-on-enhanced fluorescence and colorimetric response toward Fe^3+^ and Cu^2+^ ions, respectively and it was observed to be insensitive toward various metal ions. The Job plots revealed that the binding stoichiometry of L and metal ions is 2 : 1. In addition, density functional theory (DFT) results strongly suggested that L can be used as a powerful colorimetric sensor for the detection of Cu^2+^ ions. *In vitro* antimicrobial activities of L were evaluated by disk diffusion and results revealed good antibacterial activities against *E. coli*. Further, molecular docking was executed with DNA gyrase (PDB ID: 1KZN) of *E. coli* and the calculated interaction energy value was found to be −7.7 kcal mol^−1^. Finally, molecular docking, fluorescence, colorimetry and the HOMO–LUMO energy gap of the compound can provide new insights into developing drugs and detecting metals in biomolecules.

## Abstract 2

A novel molecule 5-bromo-2-hydroxy-*N*′-[(1*E*)-1-(thiophen-2-yl)ethylidene]benzohydrazide (L) has been designed and synthesized. The sensing studies of L showed a ratio metric & turn-on-enhanced fluorescence and colorimetric response toward Fe^3+^ and Cu^2+^ ions respectively.

## Graphical abstract

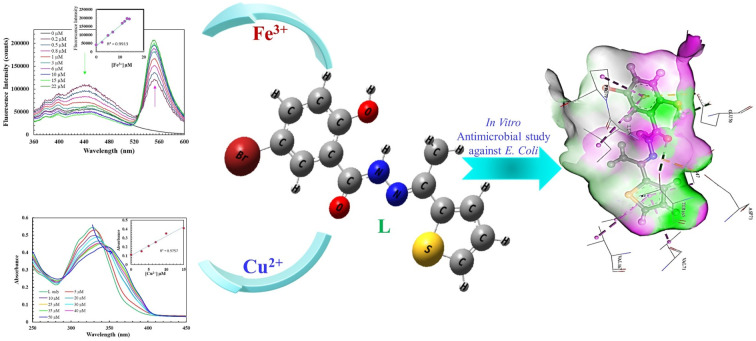

## 1. Introduction

Hydrazide–hydrazones are a unique category of Schiff base compounds containing the –CO–NHN=CH– functional group. Due to various medicinal properties such as antibacterial, antiviral, antifungal, anticancer and anti-inflammatory activities, such compounds have attracted substantial interest over many years in the design of novel chemical compounds.^1–7^ Moussa and co-workers reported a series of organic compounds containing the hydrazone moiety, which demonstrated high antioxidant activity against tyrosinase and cholinesterase.^8^ Rahim *et al.* designed and synthesized an aromatic hydrazide-based Schiff base targeting Alzheimer's disease that was shown to significantly inhibit acetylcholinesterase and butyrylcholinesterase compared to the standard physostigmine.^9^

Moreover, chemosensors containing hydrazone–hydrazide moieties are considered potential candidates due to their high sensitivity and selectivity for detecting several cations and anions.^10^ Fluorescent sensors have attracted extensive attention from researchers in the past few decades owing to their operational simplicity, high sensitivity, extreme selectivity, low instrumental cost, nondestructive sample analysis, *etc.*^11^ The signal of a fluorescent sensor is usually monitored as a change in its emission intensity, fluorescence lifetime, or a shift of emission wavelength to subtle environmental changes. On the other hand, the development of highly selective ratio metric and “turn-on” fluorescent probes have superior significance due to their capability of detecting metal ions even in living systems, which was found to display different emission behavior upon metal binding.^12,13^

Iron is one of the most abundant metals in the human body as well as in the Earth's crust.^14^ It plays a ubiquitous role in many biochemical processes of plants and animals including oxygen transport, cellular metabolism, electron transfer and serves as an active site in myoglobin, hemoglobin, siderophores, and cytochromes.^15^ Especially, ferric ion (Fe^3+^) is responsible either for structural purposes or as part of a catalytic site in many proteins, lipids and enzymes.^16^ The deficiency of iron will lead to low oxygen delivery to cells, resulting in anemia, hemochromatosis, liver/kidney damage, diabetes, and cancer.^17^ Equally, excess iron in a living cell is considered a biohazard as it is prone to produce reactive oxygen species (ROS) through Fenton-reaction, which induces several serious diseases, such as Alzheimer's, Huntington's, and Parkinson's diseases.^18^ On the other hand, Cu^2+^ is the third most essential transition metal in the human body, which plays several important roles, for example, iron absorption, maintaining nerves, and blood vessels.^19^ Moreover, it acts as a catalytical co-factor in several metalloenzymes, for instance, superoxide dismutase, cytochrome c oxidase, and tyrosinase.^20^ However, the abnormal level of copper intake also causes destructive consequences to the body (WHO >2 ppm), such as irritation of the nose and throat, nausea, vomiting, and diarrhoea.^21,22^ On the contrary, copper deficiency in the human body leads to abnormal growth of bones. It turns toxic due to accumulation in cell membranes that lead to the disorder of cellular homeostasis, which causes oxidative stress accompanied by several neurodegenerative syndromes, ranging from Menkes, Wilson, hallucinations, depression, familial amyotrophic lateral sclerosis, schizophrenia, and Alzheimer's.^23,24^ Hence, the design and synthesis of new chromo-fluorogenic sensors for the detection of metals through simple synthetic mechanisms are of considerable importance for the environment and human health.

**Scheme 1:**
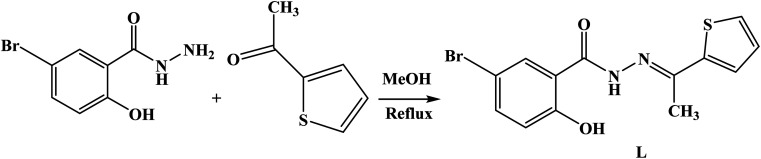
Synthesis of L from 5-bromo-2-hydroxybenzohydrazide and 2-acetylthiophene in MeOH.

Therefore, the present study was designed to synthesize hydrazide–hydrazone-based compound L derived from the coupling of aromatic ketone and phenolic acids hydrazides containing bromo and hydroxyl groups. L possesses a tridentate –ONS– binding pocket (Scheme 1). Density functional theory (DFT) calculations were performed to check the geometrical and electronic structural features of L, Fe^3+^ and Cu^2+^ complexes. In addition to that, L was tested *in vitro* antimicrobial study against some Gram-negative, Gram-positive bacteria, and the fungus strain showed moderate inhibitory results. Finally, molecular docking methodology was used to study the molecular behavior of L with *E. coli* to identify their binding interactions.

## 2. Experimental data

All chemicals were purchased from Sigma-Aldrich and used as received without further purification. Infrared (IR) spectra were recorded on a Shimadzu (FTIR) Prestige-21 spectrophotometer (range: 4000–400 cm^−1^, using KBr disk), ^1^H, and ^13^C NMR spectra were recorded on a Bruker DPX-400 spectrophotometer using tetramethyl silane as an internal reference. NMR spectra were recorded on dimethyl sulfoxide (DMSO) solvent. Mass spectra were obtained from VG Micro mass 7070HS (EI) and HP1100MSD (LCMS) spectrometers. Steady-state fluorescence spectra were recorded on a HORIBA NanoLog spectrofluorometer. Time-correlated single photon counting (TCSPC) measurements using a HORIBA-Deltaflex were conducted for fluorescence lifetime measurements.

### 2.1. Synthesis of L

The synthetic procedure for the new sensor is described as follows. A solution of 5-bromo-2-hydroxybenzohydrazide (0.5 g, 3 mmol) in methanol (20 mL) was added to a solution of 2-acetyl thiophene (0.83 g, 2 mmol) in methanol (10 mL) with continuous stirring. The resulting mixtures were stirred at 60 °C over a period of 6 hours (Scheme 1). A yellow precipitate was formed during the reaction which was collected by filtration and dried under a vacuum. Yield: 47% mp: >240 °C. IR (KBr, cm^−1^) (Fig. S1†): *ν* 3477s (–OH), 3290s (–NH), 1637s (–C=O), 1599s (–C=N), 956s (–N–N). ^1^H NMR (DMSO, 400 MHz) (Fig. S2†): *δ* 12.11 (s, H, phenolic OH), 11.25 (s, H, NH), 8.03 (s, H, H–phenyl), 7.65 (d, H, *J* = 2.4 Hz, H–phenyl), 7.59 (d, H, *J* = 2.0 Hz, H–phenyl), 7.57 (d, H, *J* = 2.8 Hz, H–thiophene), 7.13 (dd, H, *J* = 3.2 Hz, H–thiophene), 7.00 (d, H, *J* = 5.8 Hz, H–thiophene), 2.37 (s, 3H, H–methyl). ^13^C NMR (DMSO, 100 MHz) (Fig. S3†): *δ* 176.86, 160.85, 156.31, 143.34, 136.11, 132.87, 129.68, 128.94, 128.09, 120.62, 119.78, 108.09, 14.75. ESI-MS: calculated for [L]^+^ (Fig. S4†): *m*/*z* 337.9725. Found: *m*/*z* 337.9712.

### 2.2. Antimicrobial activity assay

*In vitro* antimicrobial action of compound L was assessed by agar disc diffusion technique.^1^ Mueller Hinton Agar (MHA) media was utilized as a control for bacteria analysis and Potato Dextrose Agar (PDA) media was utilized for fungal strain as already discussed and reported earlier.^1^ After preparation, the MHA and PDA media were nurtured for a day, and contaminations were tested. The test organism was inoculated by means of the sterile cotton bar on media after incubation. The sample discs were kept for 1 day for antibacterial assay at 37 °C and for 2 days at 26 °C for antifungal assay on pre-inoculated agar plates and aerobically incubated. DMSO was employed as a control. Each disc was introduced with 50 μL of the sample in DMSO having 300 μg of the synthesized compound, L. 50 μg per disc for the standard ceftriaxone and amphotericin B solutions in DMSO were loaded per disc for antibacterial and antifungal assays as a positive control, respectively. The diameter of the zone of inhibition (ZOI) in mm circling of the disc was assessed after the timely incubation for antibacterial and antifungal assay. In this research, two Gram-positive *Staphylococcus aureus* (cars-2) and *Bacillus megaterium* (BTCC-18), two Gram-negative *Escherichia coli* (carsgn-2) and *Salmonella typhi* (JCM-1652) bacteria, as well as two fungal strains *Trichoderma harzianum* (carsm-2) and *Aspergillus niger* (carsm-3) were utilized.

### 2.3. Protein–ligand docking

The 3D crystal structure of *E. coli* (PDB ID: 1KZN) was obtained in pdb format from the online protein data bank (PDB) database. The structure was verified, and energy minimization was performed with the Swiss-Pdb Viewer software packages (version 4.1.0),^25^ since the crystal structure contains a variety of issues related to improper bond order, side chains geometry, and missing hydrogen atoms. Prior to docking, all the heteroatoms and water molecules were removed from the crystal structure using PyMol (version 1.3) software packages.^26^ Both the structures of the proteins and ligands were saved in .pdbqt format by PyRx 0.8 for docking analysis.^27^ The grid box that encloses amino acids domain involved in the binding active sites, had the dimension of 25 × 25 × 25 Å^3^ (x × y × z) and center of 19.53 × 19.16 × 43.28 Å^3^ (x × y × z). The docked conformation of the respective protein conformer with the lowest binding free energy was analyzed using PyMOL Molecular Graphics System (version 1.7.4) and Accelrys Discovery Studio 4.1.^28^

## 3. Results and discussion

### 3.1. Colorimetric analysis of Cu^2+^

**Fig. 1:**
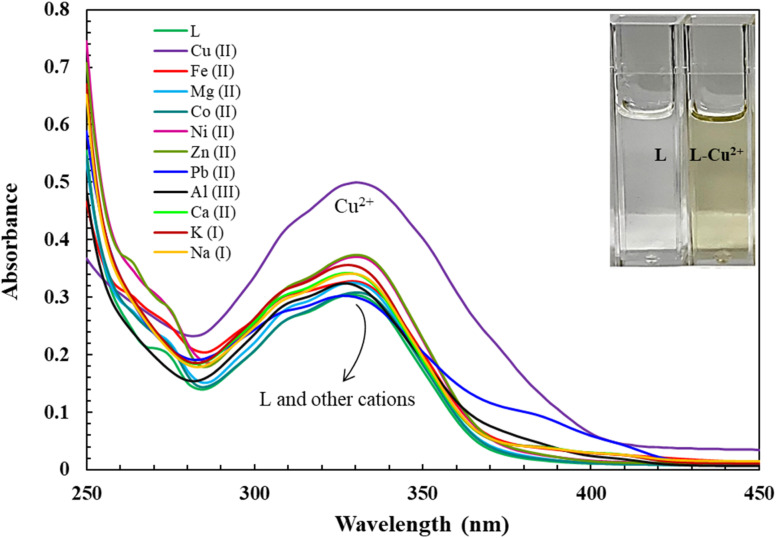
Absorbance *vs.* wavelength (nm) of ligand L (10 μM) with equivalent addition of different metal ions in DMSO. Inset: visual color changes of L and L-Cu^2+^ under daylight.

**Fig. 2:**
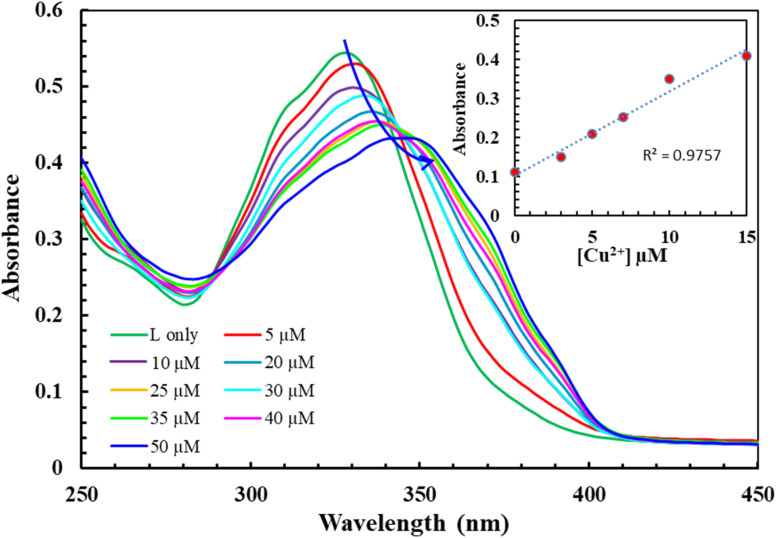
Absorbance *vs.* wavelength (nm) of L (10 μM) in the presence of varying concentration of Cu^2+^ (0–100 μM) in DMSO. Inset: detection limit of L (10 μM) towards Cu^2+^ based on 3*σ*/slope at 346 nm using UV-visible titration experiment.

The recognition profiles of L (10 μM) toward different metal cations (1 eq.) were also investigated by absorption spectroscopy. The selectivity of L towards various cations Cr^3+^, Mn^2+^, Pb^2+^, Fe^2+^, Fe^3+^, Co^2+^, Ni^2+^, Cu^2+^, Zn^2+^, Na^+^, K^+^, Mg^2+^, Ca^2+^, and Al^3+^ was achieved in DMSO as demonstrated in Fig. 1. When an equivalent amount of the cations was added to the L, only Cu^2+^ enhanced significantly produced an intensive color change, while the rest of the metal ion solutions showed a very mild alteration or almost no change in color. Hence, the combination of the ligand with copper ions was further investigated by spectroscopic and theoretical means. The UV-vis absorption spectra of the ligand in DMSO showed an absorbance maximum at 330 nm due to a symmetry-allowed π → π* transition rather than a symmetry-forbidden n → π* transition. In addition, two isosbestic points were observed at 290 and 346 nm in the colorimetric titration of the ligand with Cu^2+^ as shown in Fig. 2, which clearly indicates the smooth molecular conversion of free ligand to metal complex. The detection limit of L toward Cu^2+^ was obtained using the calibration curve of absorbance *versus* concentration, which was found to be as low as 2.35 μM.

### 3.2. Fluorescence detection of Fe^3+^

**Fig. 3:**
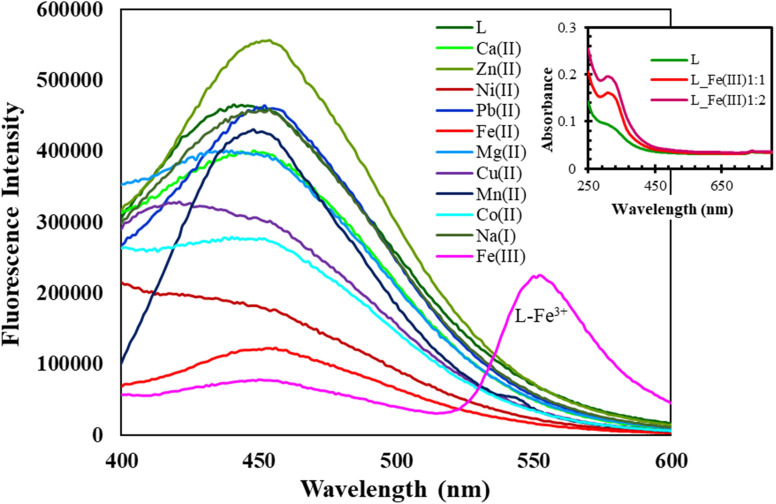
Fluorescent variations of L (10 μM) with various cations (1.0 equiv.) with an excitation at 360 nm; inset: comparison of UV-vis spectra of L (10 μM) before and after addition of Fe^3+^.

**Fig. 4:**
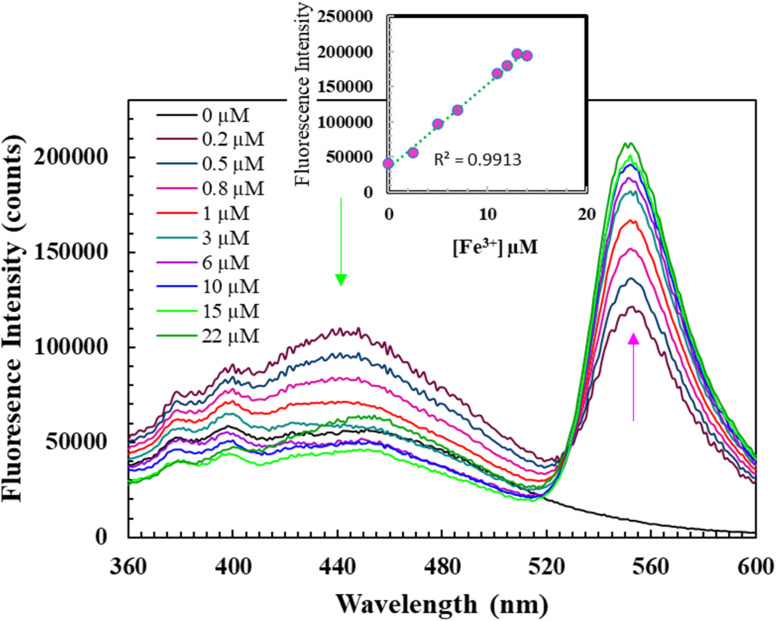
Fluorescence spectra of L (10 μM) (*λ*_ex_ = 360 nm) with varying concentrations of Fe^3+^ in DMSO; insets: detection limit of L (10 μM) towards Fe^3+^ based on 3*σ*/slope at 550 nm using fluorometric titration experiment.

Fig. 3 illustrates the fluorescent alterations of L (10 μM) with diverse cations (1.0 equiv.) with an excitation at 360 nm whereas, the inset shows a comparison of UV-vis spectra of L (10 μM) before and after addition of Fe^3+^. As evident from Fig. 4, the DMSO solution of L (10 μM) shows fluorescence emission at 455 nm when excited at 360 nm. Upon the addition of 1 eq. of Fe^3+^, a new significant broad fluorescence emission at about 550 nm was clearly observed, whereas Cr^3+^, Mn^2+^, Pb^2+^, Fe^2+^, Fe^3+^, Co^2+^, Ni^2+^, Cu^2+^, Zn^2+^, Na^+^, K^+^, Mg^2+^, Ca^2+^, Al^3+^ and Bi^2+^ showed no emission band at this wavelength. These results indicated that L could function as a fluorescent “turn-on” type sensor for Fe^3+^ ions. It can be assumed that the emergence of the new fluorescence emission might demonstrate the coordination process of Fe^3+^ with L, which is also supported by the generation of a new absorption band at 355 nm in the UV-vis spectrum of L + Fe^3+^ when compared with that of L (Fig. 4; Inset). In addition, the remarkable stokes shift (*λ*_em_ − *λ*_abs/ex_ = 195 nm) of L + Fe^3+^ enables the clear separation of the excitation and emission bands, which is highly desirable for the fluorescent detection of metals. For better investigation, we have carried out the fluorescence titration of L with the subsequent addition of Fe^3+^ and found that the emission intensity at 550 nm gradually increased. The ratio metric and fluorescence enhancement of L witnessed the visual detection of Fe^3+^ ions when the samples were exposed to a UV lamp. The quantum yields of L increased from 0.0019 to 0.052 upon binding of Fe^3+^. Accordingly, L could be utilized as an excellent turn-on fluorescence chemoreceptor for Fe^3+^ ions (Fig. 4). The detection limit of L toward Fe^3+^ was obtained using the calibration curve of emission *versus* composition, which is an important factor for a better sensor. According to the U.S. EPA regulations, it should be less than the limit of ∼20 μM or less.^29^ The detection limit of L toward Fe^3+^ ions was found to be as low as 3.87 μM.

**Fig. 5:**
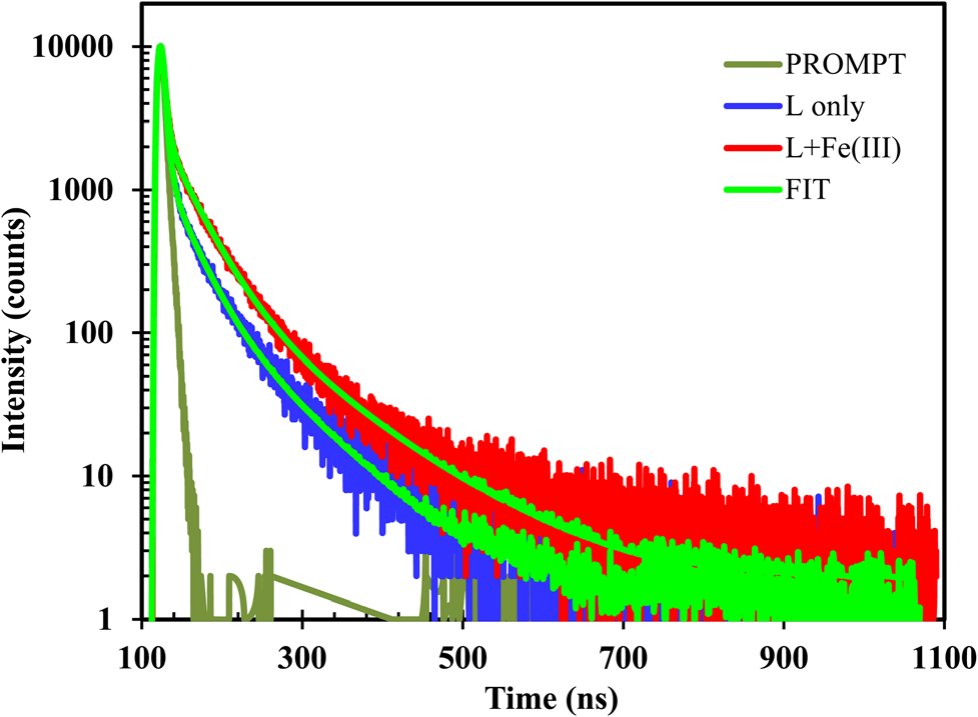
Excited state fluorescence decay behavior of L and complexes with Fe^3+^ (10 μM) in DMSO solvent at room temperature (excitation wavelength: 400 nm).

The photophysical properties of L were studied using time-resolved emission experiments in the absence and presence of Fe^3+^. Lifetime experiments for the chemosensors L and its Fe^3+^ complex was studied at 298 K in DMSO solvent. The values of fluorescence lifetime of L and L + Fe^3+^ are 3.46, and 6.57 ns, respectively as represented in Fig. 5. Both L and its complex showed a bi-exponential decay, which may be due to the formation of different hydrogen-bonded species in highly polar DMSO solvents.^30^

### 3.3. Interaction mode and sensing mechanism

**Fig. 6:**
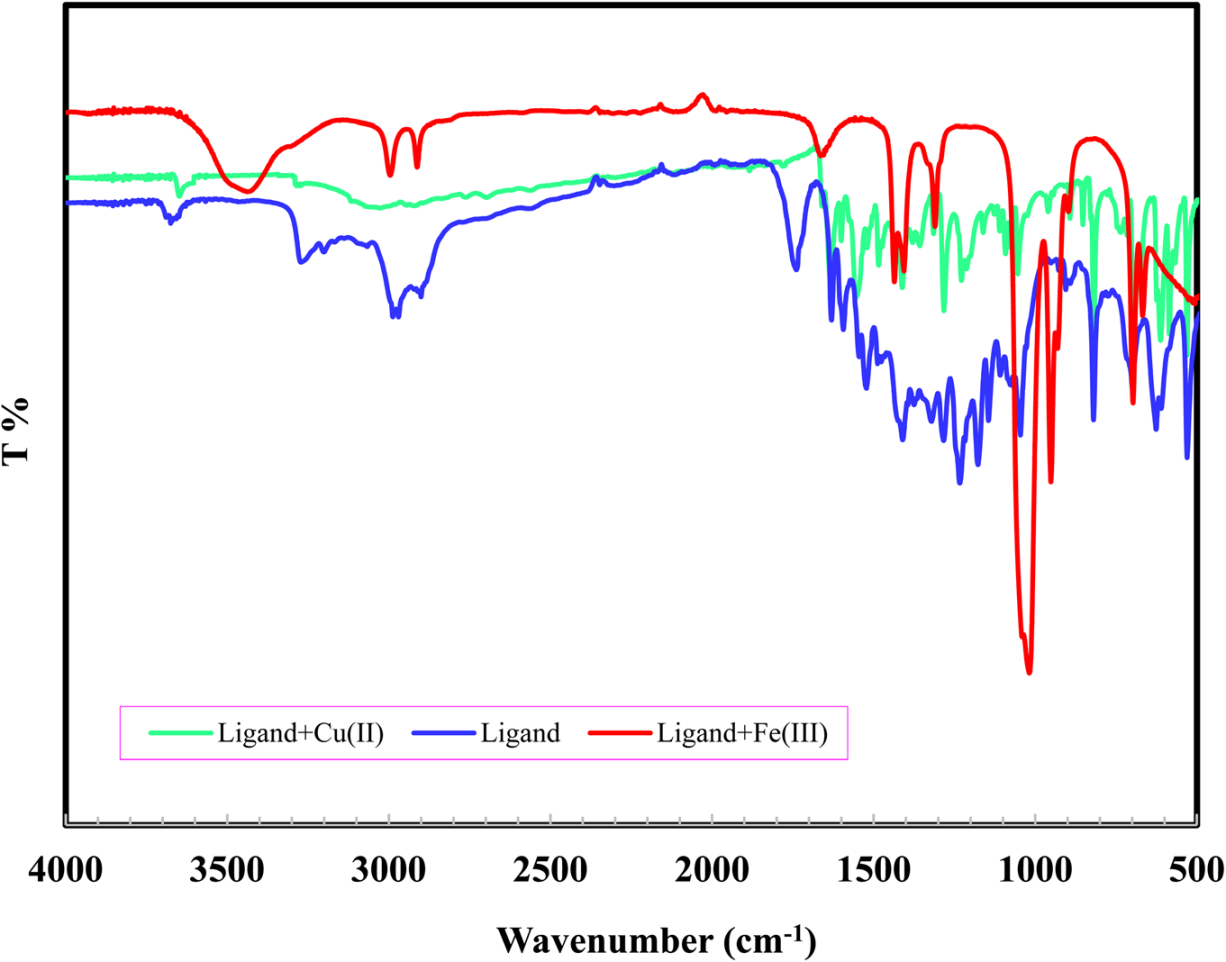
FT-IR spectra of L and, its Fe^3+^ and Cu^2+^ complexes recorded in KBr.

To find out the interaction mode of Fe^3+^ and Cu^2+^ with L, the Job's plot and FT-IR were employed. The stoichiometric ratio between Fe^3+^ and Cu^2+^ with L was established through the continuous variation method. From Job's plot, it is evident that the ratio between the ratio of Fe^3+^ and Cu^2+^ with L was 1 : 2 (see ESI Fig. S5 and S6†). To understand the binding sites between them further, IR spectra were recorded in KBr as shown in Fig. 6. From the IR spectra of L, L + Cu^2+^ and L + Fe^3+^, it is distinct that the stretching vibration band of –C=O (1637 cm^−1^) disappeared with the addition of metals (Fig. 6). This suggested that the coordination has taken place through an oxygen atom of C=O group. At the same time, shifting of –C=N stretching vibration peak to lower wave number in the complex from 1599 to 1586 cm^−1^ suggested the involvement of azomethane nitrogen in bonding.^31^ The bands at 3400–3600 cm^−1^ in L, Cu^2+^ and Fe^3+^ complexes, are assigned to phenolic–OH.^32^

**Fig. 7:**
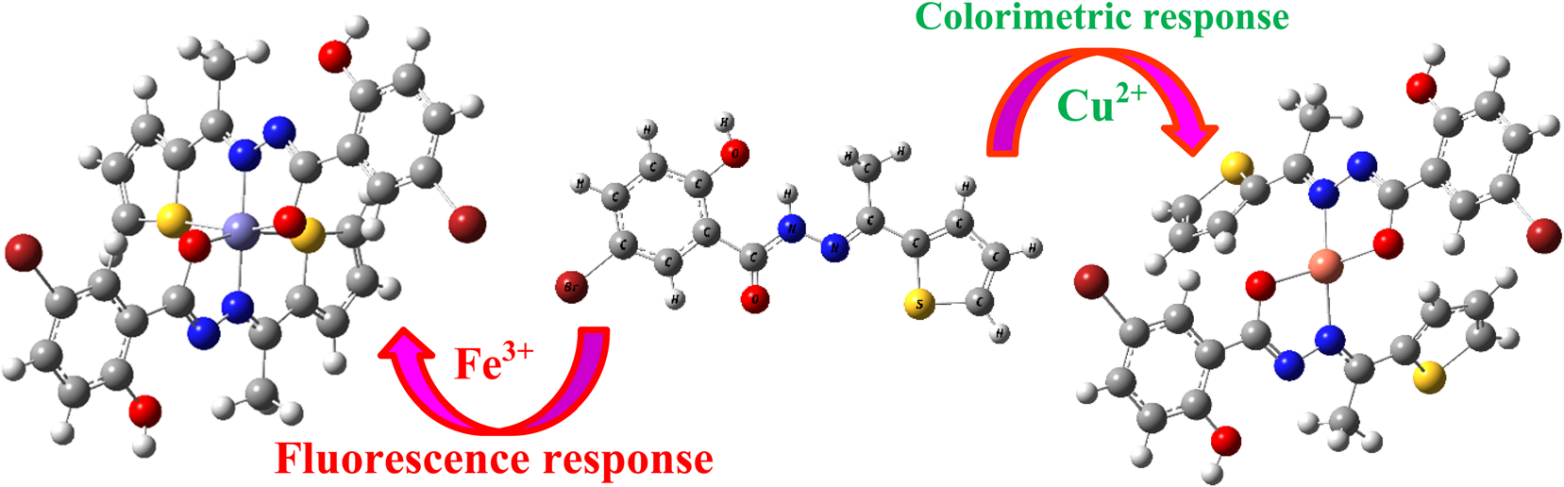
Plausible binding mode for L in upon addition of Fe^3+^ and Cu^2+^.

Fig. 7 illustrates the possible binding mode for L in upon addition of Fe^3+^ and Cu^2+^. It seems that Fe^3+^ and Cu^2+^ adopts a coordination mode with nitrogen atoms (–C=N) of the receptor L, that inhibits the non-radiative transitions such as –C=N isomerization. The –C=N isomerization inhibition process induced by the Fe^3+^ binding warrants the chelation-induced enhanced fluorescence (CHEF) effect.^33^ Thus, the fluorescence enhancement of L upon binding to Fe^3+^ might be due to CHEF effect.

### 3.4. Application of L in water sample

To test the reliability of L in a real sample with Cu^2+^ ion, it was employed to detect concentration of Cu^2+^ in tap water samples. In order to verify the accuracy of the established procedure, recovery experiments were carried out by spiking the tap water samples with different concentrations of Cu^2+^ and L in DMSO (2 mL) before any pretreatment.^34^ The change in absorbance of L with the above water samples was tested by adding 20, 40, and 80 μM of Cu^2+^ individually. Each sample was analyzed three times in H_2_O/DMSO (v/v, 8/2). The results are tabulated in Table 1. The results suggest that sensor L can be used in real test samples.

**Table 1:** Application of L for the determination of Cu^2+^ concentrations in tap water samples

Sample	Added Cu^2+^ (μM)	Found (μM)	Recovery (%)	RSD (%) (*n* = 3)
Tap water	20	15.4	77	1.3
	40	31.6	79	1.2
	80	61.1	76	1.5

### 3.5. Antimicrobial study

*In vitro* sensitivities of two Gram-positive and two Gram-negative bacteria including two fungal strains against the synthesized compound were assessed by agar disc diffusion technique.^1,35^ The formation of the diameter of ZOIs in mm by the synthesized analogue is shown in Table 2. Compound L demonstrated moderate activity against *E. coli* bacteria compared to other bacterial and fungal strains. The outcomes represented in Table 2 point out that L had better antibacterial activity against *E. coli*, with a mean ZOI of 12 mm diameter at 300 μg mL^−1^. While, L had a low mean ZOI for *T. harziana*, and *A. niger*, suggesting that had a low antifungal activity effect.

**Table 2:** Diameter of inhibition zones (mm) of the synthesized compounds ceftriaxone, and amphotericin B against tested bacterial and fungal strains

Compounds	Gram (+) bacteria		Gram (−) bacteria		Fungi	
	*S. aureus*	*B. megatherium*	*E. coli*	*S. typhi*	*T. harzianum*	*A. niger*
L	9	9	12	10	7	6
Ceftriaxone	40	50	38	44	—	—
Amphotericin B	—	—	—	—	8	17
DMSO	—	—	—	—	—	—

### 3.6. Molecular docking analysis

Molecular docking, a subfield of computational chemistry and bioscience, is a powerful tool to investigate and provide several quick insights into ligand–receptor interactions in order to facilitate the design of potential drugs.^36,37^

**Fig. 8:**
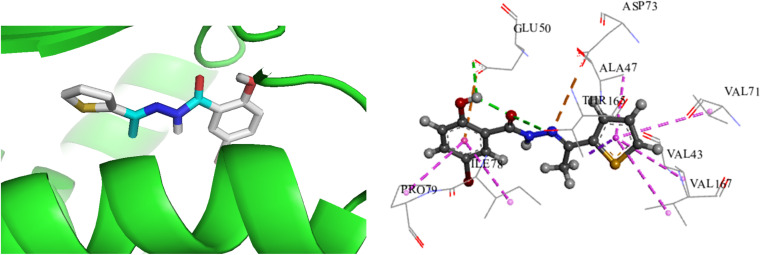
Binding mode of L docked with *E. coli* DNA gyrase (PDB ID: 1 KZN); [green: H-bond, pink: hydrophobic interactions].

To investigate and compare the antimicrobial activity of the synthesized compound with experimental data, a docking analysis of L against *E. coli* was performed. Thus, to get the binding conformations of these compounds, L was docked in the active site of *E. coli* DNA Gyrase (PDB ID: 1KZN) using the PyRx 0.8. The most stable anchoring conformations of these compounds along with interacting residues are shown in Fig. 8 created with the help of the discovery studio visualizer.^29^

**Fig. 9:**
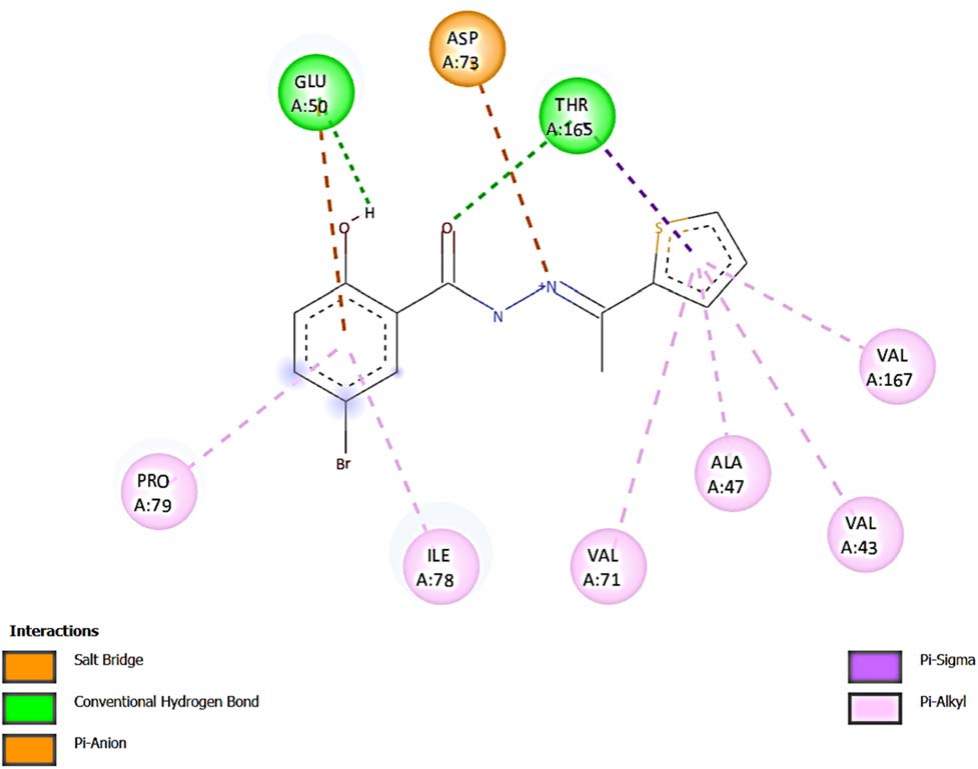
2D diagram for the interaction mode of L with amino acid residues of *E. coli* DNA gyrase (PDB ID: 1 KZN).

The binding energies for L with 1KZN were −7.7 kcal mol^−1^, whereas the binding energy for Ciprofloxacin was reported −9.2 kcal mol^−1^.^38^ In L-1KZN, L formed two conventional hydrogen bonds (2.63 and 3.00 Å) of O–H–O–C with active site residues GLU50 and THR165. Pi-anion, pi-sigma and pi-alkyl bonds were also noticed with GLU50 (4.23 Å), VAL43 (4.62 Å), ALA47 (5.43 Å), VAL71 (5.47 Å), ILE78 (5.07 Å), VAL167 (5.06 Å), PRO79 (4.82 Å), and THR165 (3.75 Å) respectively. It was also noted that an electrostatic bond was found between N and residue ASP73 as shown in Fig. 8. Results of docking studies revealed that L formed bonds to the active site of 1KZN and showed strong interactions with GLU50, THR165, and ASP73 of DNA gyrase enzyme (PDB ID: 1KZN) as shown in Fig. 9, which also supports the literature.^20,39^ Thus, computational results are in good agreement with *in vitro* experimental data (Table 3).

**Table 3:** Calculated binding energies and H-bond count of L in the active site of *E. coli* DNA Gyrase (PDB ID: 1KZN)

System	Binding energy (kcal mol^−1^)	L-Protein interaction			
		No of H-bond	Amino acid residues	Distance (Å)	Other interacting residues (distance)
L-1KZN	−7.7	2	GLU50 THR165	2.63 3.00	THR165 (3.75 Å), GLU50 (4.23 Å), VAL43 (4.62 Å), PRO79 (4.82 Å), VAL167 (5.06 Å), ILE78 (5.07 Å), ALA47 (5.43 Å), VAL71 (5.47 Å)

### 3.7. Computational analysis of frontier molecular orbitals

To get an insight into the change of the energy gap of L before or after interaction with metal ions, we carried out the quantum mechanical calculations of the free probe and its metal coordinated complexes with the help of the DFT method using a Gaussian 09 program at the level of B3LYP/6-311G(d)/LANL2DZ for C, H, N, S, O, and metal ions, where for the complexes multiplicity were 2.

**Fig. 10:**
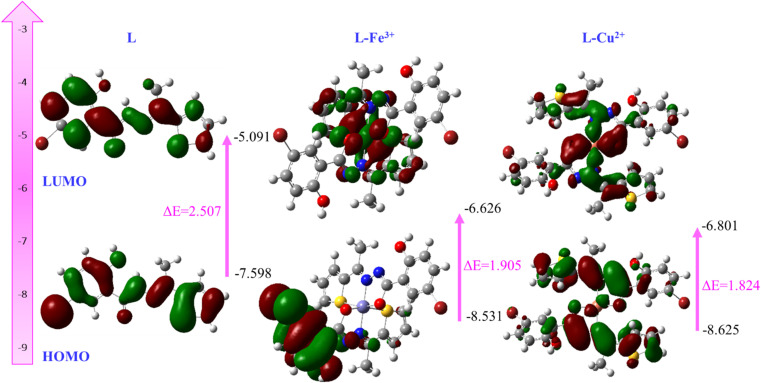
Frontier molecular orbitals of L, L-Fe^3+^ and L-Cu^2+^ generated by B3LYP/6311G(d)/LANL2DZ.

The frontier molecular orbitals provided effective information about electronic transitions, reactivity, biological activity, and kinetic stability of L as well as metal complexes of Fe^3+^ and Cu^2+^.^40–43^ The highest occupied molecular orbital (HOMO) and lowest unoccupied molecular orbital (LUMO) of L were located within mostly on the whole π-moiety of thiophene as well as the –CO–NHN=CH– moiety with a HOMO–LUMO gap of 2.507 eV, which indicated the transition of π to π* of the thiophene unit. As evident from UV-visible and fluorescence titration spectra, the stoichiometry of L with Fe^3+^ and Cu^2+^ were confirmed strongly as 2 : 1 and hence, the optimization of the metal complex was carried out with 2 : 1 coordinated complexes. In the L-Fe^3+^ complex, the HOMO is situated completely on the whole ligand L, whereas LUMO is concentrated over Fe^3+^ ions with a band gap of 1.095 eV (Fig. 10). These results clearly revealed the interruption of internal charge transfer after the addition of Fe^3+^ ions to L. Similarly, the calculated HOMO–LUMO gap of Cu^2+^ complex was small compared to that of L. These findings intensely supported the reason for the colorimetric response upon the addition of metal ions Cu^2+^ to L.

Moreover, Snyder *et al.* reported that the smaller energy gap between HOMO and LUMO is a pivotal determinant to predict drug–receptor interactions and their electronic configuration.^44^ Thus, the smaller energy gap of L supports antimicrobial activity.

## 4. Conclusion

In summary, a novel hydrazide–hydrazone-based chemosensor has been synthesized and characterized successfully by FT-IR, ^1^H and ^13^C NMR and mass spectroscopy. The antimicrobial assay revealed significant inhibition of L against *E. coli* Gram-negative bacteria. Fluorescence studies revealed that there was an enhanced fluorescence in the emission spectra, upon the addition of Fe^3+^ ions and that makes L a potential ratio metric as well as fluorescence turn-on chemosensor, which was strongly supported by the elevating quantum yields. Further, the novel sensor exhibited colorimetric responses with significant color change from colorless to yellow toward biologically important metal ion Cu^2+^ in DMSO over other metal cations ions (Cr^3+^, Mn^2+^, Pb^2+^, Fe^2+^, Fe^3+^, Co^2+^, Ni^2+^, Zn^2+^, Mg^2+^, Ca^2+^, Al^3+^ and Bi^2+^). That's why L can act as an excellent chemosensor for the detection of Cu^2+^ in the environment. Also, L may be applied for the detection of biologically important metal ion Fe^3+^ in the bioimaging of live cells. Thus, due to the wide application of such hydrazide–hydrazone compounds and their simple, low-cost synthetic procedure, such compounds can be a target for drug design.

## Author contributions

Conceptualization, M. A. M. P. and S. A. R.; methodology, M. A. M. P., and S.A. R.; software, S. A. R. and M. A. M. P.; validation, A. K. M. R. U., M. A. M. P. and J. U.; formal analysis, M. A. M. P., S. A. R. and W. E. G.; investigation, S. A. R., W. E. G., A. A. and M. A. H.; resources, M. A. M. P., M. A. H., M. K. and J. U.; data curation, M. A. M. P., W. E. G., A. A. and S. A. R.; writing – original draft preparation, S. A. R., M. A. M. P. and A. K. M. R. U.; writing – review and editing, all authors; visualization, M. A. M. P., M. K. and J. U.; supervision, M. A. M. P. and J. U. All authors have read and agreed to the published version of the manuscript.

## Conflicts of interest

The authors declare no competing financial interest.

## Supplementary Material

RA-013-D3RA04364A-s001
