# Supplementary material for: Synthesis of a novel hydrazone-based compound applied as a fluorescence turn-on chemosensor for iron(iii) and a colorimetric sensor for copper(ii) with antimicrobial, DFT and molecular docking studies
Source: RSC Adv. 2023 Aug 9;13(34):23819–28. doi: 10.1039/d3ra04364a (PMC10411390; doi:10.1039/d3ra04364a)
Supplement: RA-013-D3RA04364A-s001 [file RA-013-D3RA04364A-s001.pdf]

Electronic Supplementary Material (ESI) for RSC Advances.
This journal is © The Royal Society of Chemistry 2023

Synthesis, DFT, Antimicrobial and Molecular docking studies of Novel thiophene hydrazone ligand with Fluorescence turn-on response of Fe(III) and colorimetric detection of Cu(II).

Sharmin Akther Rupa[1], Md Abdul Majed Patwary[1,], William Emmanuel Ghann[2], Adams Abdullahi[2], A. K. M. Royhan Uddin[1],* Md. Mayez Mahmud[3], Md. Aminul Haque[4], *Jamal Uddin[2,*]*

[1]Department of Chemistry, Comilla University, Cumilla-3506, Bangladesh.

[2]Center for Nanotechnology, Department of Natural Sciences, Coppin State University, Baltimore, MD, USA.

[3]Tokushima University, Faculty of Pharmaceutical Science, Tokushima Shi 770-0026, Japan

[4]Department of Chemistry, Jagannath University, Dhaka-1100, Bangladesh

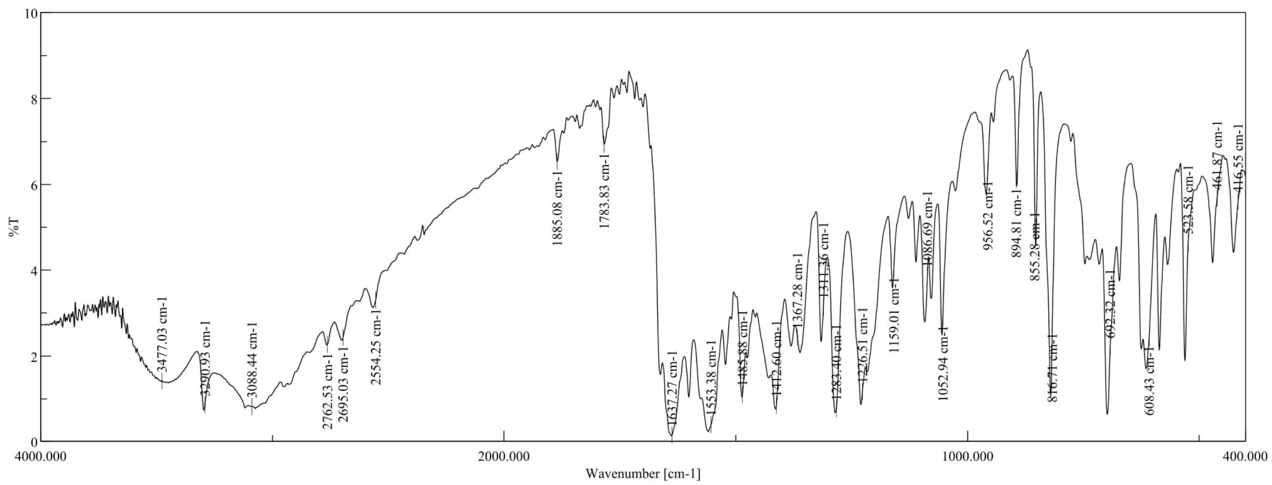

Fig. S1. FT-IR of **L**.

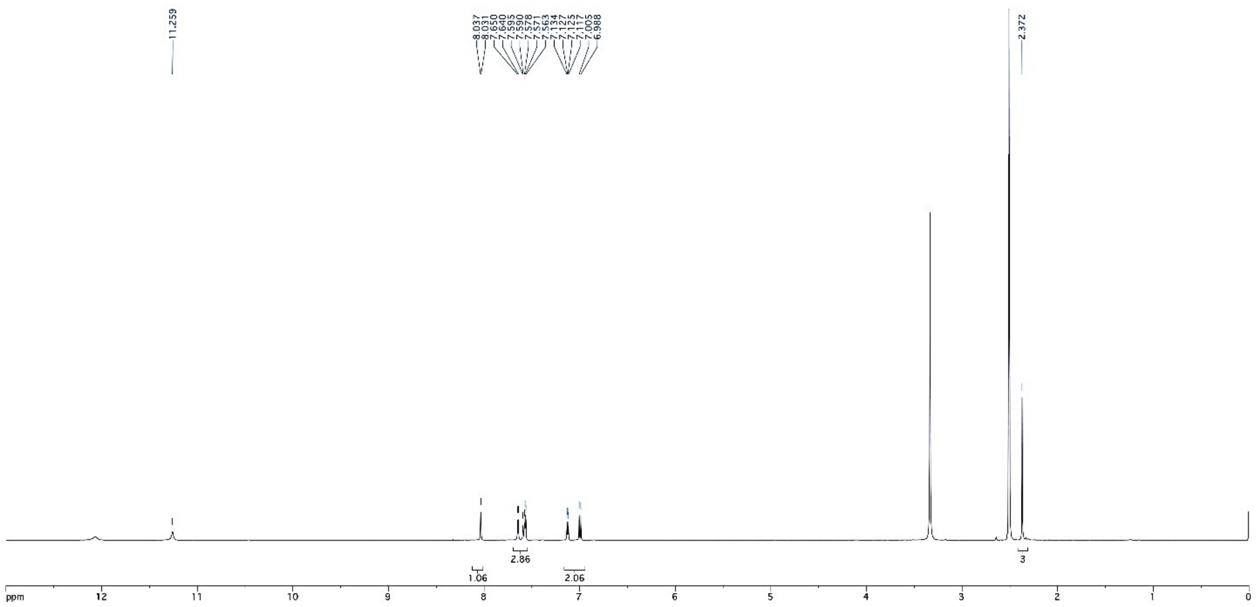

Fig. S2. ^{1}H NMR of **L**.

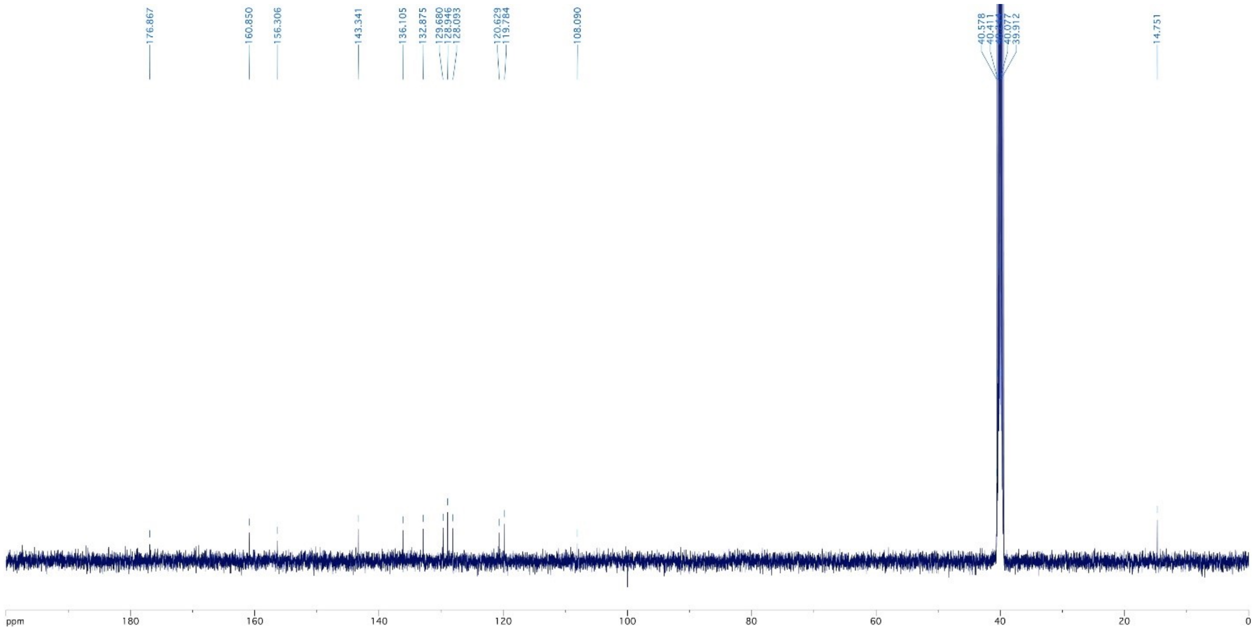

Fig. S3. ^{13}C NMR of **L**.

Elemental Composition Report

Tolerance = 100.0 mDa / DBE: min = -1.5, max = 50.0
Selected filters: None

Monoisotopic Mass, Even Electron Ions
1 formula(e) evaluated with 0 results within limits (all results (up to 1000) for each mass)
Elements Used:
C: 13-13 H: 11-11 N: 2-2 O: 2-2 P: 1-1 S: 1-1 Br: 1-1

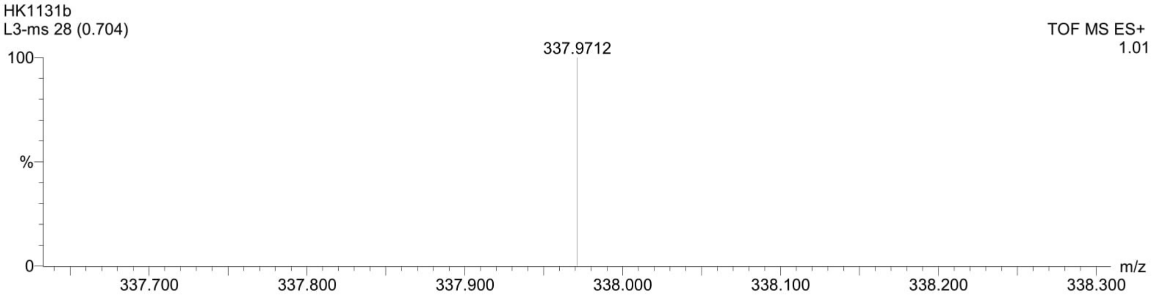

Minimum:				-1.5		
Maximum:		100.0	100.0	50.0		
Mass	Calc. Mass	mDa	PPM	DBE	i-FIT	Formula
337.9712	---					

Fig. S4. Mass Spectra of **L**.

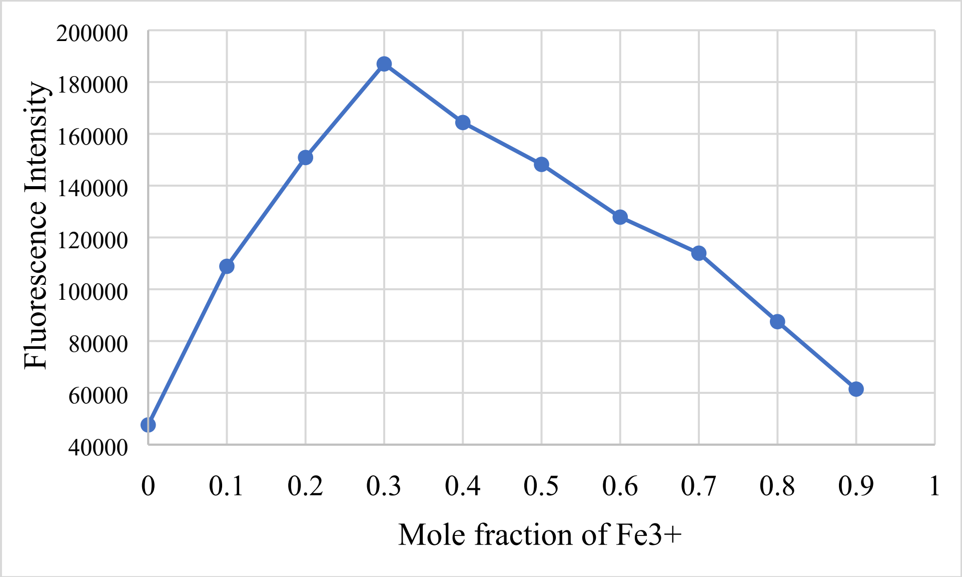

Fig. S5. Job plot for the reaction of **L** with Fe^{3+}. Fluorescent emission at 550 nm was plotted as a function of the molar ratio $[Fe^{3+}]/([\mathbf{L}]+[Fe^{3+}])$.

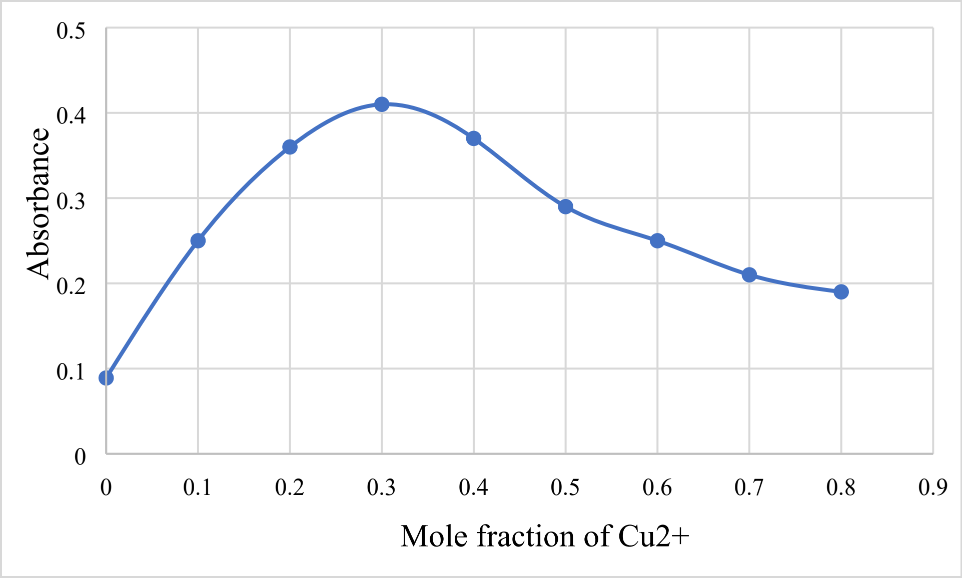

Fig. S6. Job's plot according to the method of continuous variations, indicating the 1:2 stoichiometry for L + Cu^{2+}
